# Sonarlogger: Enabling long-term underwater sonar observations

**DOI:** 10.1016/j.ohx.2024.e00531

**Published:** 2024-04-18

**Authors:** Frederik-Willem Fourie, Kobus Langedock, Roeland Develter, Harold Loop, Christopher J. Peck, Leandro Ponsoni, Hans Pirlet, Wieter Boone

**Affiliations:** a Flanders Marine Institute (VLIZ), Jacobsenstraat 1, 8400 Oostende, Belgium

**Keywords:** Coastal monitoring, Sonar, Acoustic, Long-term measurements, Raspberry Pi

## Abstract

Coastal seas are under increasing pressure from extreme weather events and sea level rise, resulting in impacts such as changing hydrodynamic conditions, coastal erosion, and marine heat waves. To monitor changes in coastal marine habitats, such as reefs and macrophytes meadows, which add to the resilience of our coasts, consistent, medium- to long-term seafloor observations are needed. This project aims to deliver repeated, high-frequency sonar surveys on a stationary seabed mooring of a specific target area over a period of up to several months. A new stand-alone subsea system, the Sonarlogger, based on a battery pack, low-power logger and a high-resolution scanning sonar, was developed. It allows for long-term deployments with a customisable battery pack, WI-FI download and configurable sleep state. The system has been tested for over 130 days in dynamic coastal environments off the Belgian coast. Combined with auxiliary sensors, such as for measuring currents, waves and turbidity, this system enables comprehensive studies of morphologic changes and changing benthic ecosystems. Moreover, this system has the capacity to provide measurements of coastal environments during storms, where conventional systems may fall short, providing insights into event-based changes of the seafloor.

## Graphical abstract

**Figure:**
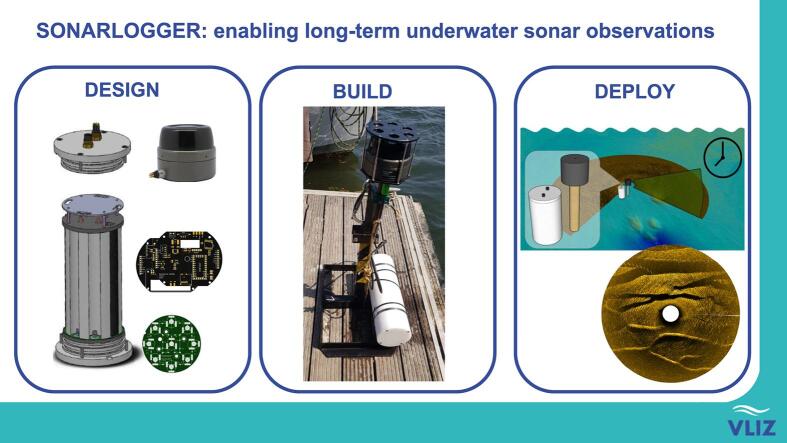

**Specifications table:** 

Hardware name	Sonarlogger
Subject area	Biological sciences Environmental, planetary and agricultural sciences General
Hardware type	Imaging tools Field measurements and sensors Logging
Closest commercial analog	Imagenex 881A Imaging sonar combined with ASL IRIS data Logger
Open-source license	CC BY 4.0
Cost of hardware	13 250 EUR including sonar, 1 700 EUR for logger, enclosure, and battery pack.
Source file repository	https://doi.org/10.17605/OSF.IO/4GTZ8

## 1 Hardware in context

Side-scan sonar is widely used in seafloor mapping [1], [2] through sporadic surveys based on vessel-mounted or towed systems which are often limited to favourable weather conditions, both in vessel capabilities and as the quality of data degrades with the increase of surface movement for such systems. This approach obscures seafloor changes occurring at relatively short time scales (e.g., tidal cycle) and during high-energetic events (e.g., storms). Among others, it concerns sediment bedform migration, coastal erosion, biomass growth and loss, gas seepage, and changes imposed by construction activities. High frequency and high-resolution measurements provide insights into how features change over time and allows these changes to be correlated to other finer scale parameters. To overcome limitations from conventional vessel-based surveys, this work introduces the Sonarlogger system that implements a stationary scanning sonar mounted on a seabed mooring, allowing for 360-degree sonar scans of the seabed continuously performed at high spatio-temporal resolution, covering a set area for periods up to several months. This aims to provide a view into changes that are not observable with a single measurement, for example determining the rate of change in the extent of fish nesting zones [3] or the evolution of sand mason worm conglomerates [4].

Specifically, the Sonarlogger system was developed and tested for observing the evolution of bivalve reefs in the North Sea. A major challenge in monitoring these reefs is the dynamic and highly turbid coastal environment in which they occur. The resulting low visibility hampers adequate visual assessments. Yet, continuous observations are necessary to gain insight into the short-term dynamics of biogenic reef development, as it enables these observed changes to be correlated with other parameters such as weather, wave action and tides. This is particularly important in the context of these reefs aiding in coastal defence through sediment stabilization and, therefore, mitigating coastal erosion which are, in turn, expected to increase under a climate changing scenario [5]. After all, it is especially during highly energetic storm events, where access to the study site is limited and underwater visibility is poor, that changes in the structure of these reefs are expected to occur. However, in these conditions, conventional seabed observation methods such as repeated vessel-based side-scan sonar surveys, time-lapse camera recordings, or diver surveys, which are visually limited to only a few meters, are not feasible. Hence, an alternative option is required. As side-scan sonars have been proven to be an effective tool for monitoring and detecting seabed habitats such as bivalve reefs [4], [6], [7], an in situ observation system was devised to keep a scanning sonar, which is far less impacted by turbidity, in the surrounds of a reef for multiple weeks to months, with high resolution scans performed over regular temporal intervals of up to 1 scan every 4 h and a maximum horizontal reach of about 60 m. To achieve this, a self-contained logger and power system was designed and built around a high-resolution commercial scanning sonar. This system was made to be deployed on a heavy (∼200 kg) seafloor lander (a frame built to land and remain stationary on the seafloor), which provided the stability to withstand the coastal environment, including storm conditions in relatively shallow waters (∼10 m).

While the system has been tested and validated for the monitoring of bivalve reefs, we do foresee a significant number of other application cases, ranging from the monitoring of other benthic habitats (e.g., macrophyte meadows) [8], [9], surveillance of hanging structures (e.g., mussel farm lines), and observation of sediment dynamics (sand ripple migration, coastal erosion, etc.). The potential application can be further improved by adding complementary sensors to the system, such as Acoustic Doppler Current Profiler (ADCP) for current and Conductivity, Temperature and Depth (CTD) sensor for temperature, salinity and depth measurements to give more context to the water masses and their behaviour during these scans.

## 2 Hardware description

### 2.1 System overview

**Fig. 1:**
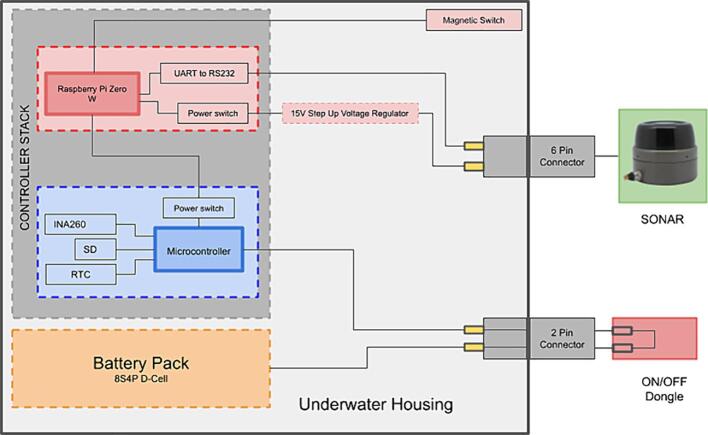
System block diagram.

**Fig. 2:**
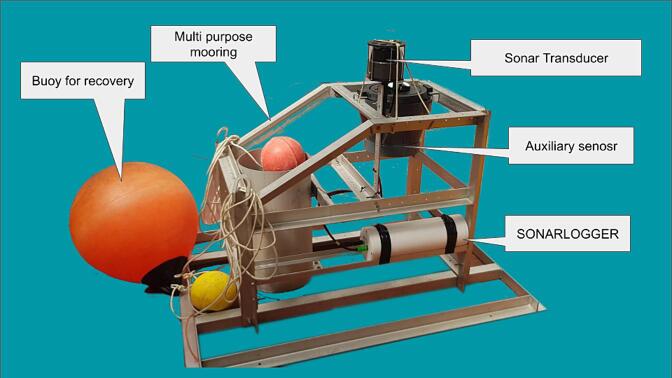
Full system in test configuration with annotations.

The presented Sonarlogger system is a custom built, long-endurance sonar imager (as seen in the block diagram in Fig. 1). Fig. 2 shows the system installed on a seafloor lander during the testing phase. The system was built as an alternative to expensive off-the-shelf high resolution sonar logging systems, as sonars as primary sensors are already costly. By putting the system to sleep between sampling intervals, the system can achieve long duration deployments. This, along with sonar itself, provides in situ observation capacity of the seafloor within a range of about 60 m, and at a high-frequency sampling for long periods. The primary components of this system are a scanning sonar, an underwater housing, a battery pack (built into the housing) and a controller with software. The components are summarised in Table 1. In the following sections, each of these components is described.

**Table 1:** Components and characteristics of the Sonarlogger system.

**Component**	**Origin**	**Function**
Scanning Sonar	Purchased	Primary sensor
Controller	Built	Data recording and power management
Battery Pack	Built	Power supply
Housing	Built	Underwater housing

**Parameter**	**Value**	**Description**

Range	60 m	Range dependent on sonar settings
Depth	500 m	Limited by the housing design parameters
Endurance	130 days	Based on 4-hour interval scans

### 2.2 Scanning sonar

To observe changes in the seafloor over time and in various marine conditions, including high-turbidity waters, a stationary and 360-degree scanning sonar was selected as the acoustic sensor. The predetermined criteria for selecting the sonar model were based on the trade-off between price, ease of integration and quality of data. The beam characteristics was deemed to be the most import in determining the quality of the data. A wide vertical beam allows for better horizontal seafloor coverage, whereas a narrow horizontal beam is needed for a higher resolution. The vertical beam width is further discussed in section 6.2.1. In the case of horizontal beam width, if two objects are less than the horizontal beamwidth apart, they cannot be distinguished from one another as they will be represented by a single return, meaning that the beam produces more useful information at a longer range. In basic trigonometry, the width of this return can be expressed as shown in Eq. (1). See Table 2 for a comparison of horizontal beam widths.

(1) $$L_{\mathrm{return}}=2\;\times \;L_{\mathrm{range}}\times \;\sin\left(\frac{\emptyset }{2}\right)$$

**Table 2:** Comparing the return widths at 10 m range of different horizontal beam widths.

**Horizontal beam width (degrees)**	**Range (m)**	**Return width (cm)**
1	10	17.453
2	10	34.900
3	10	52.353

Among the available options, the Echologger RS900 high-resolution scanning sonar attended these criteria, allowing high-frequency sampling at fine horizontal resolution with a comparatively lower price and good manufacturer support for third-party integrators. Table 3 compares the price, ease of integration and beam characteristics of a few other commercially available scanning sonars. Moreover, the selected model is suitable for long-term deployments, as it comes standard in an anodised aluminium housing and protecting guard which adds protection against corrosion and reduces damages from possible impacts.

**Table 3:** Ease of integration, beam characteristics (frequency and beam angle), and price of a few other commercially available scanning sonars.

**Sonar**	**Depth Rating**	**Logger optional?**	**Power**	**Integration Ease**	**Frequency**	**Beam Angle**	**Price (EUR)**
Echologger RS900	1000 m	No	6 W	****	900 kHz	H 0.8, V 30	11 550
Blue Robotics Ping360	300 m	No	5 W	*****	750 kHz	H 2, V 25	2 215
Imagenex 881A	1000/3000 m	Yes	5 W	**	1 MHz	H 0.9, V 10	27 760
Tritec Micron	750/3000 m	No	4 W	**	700 kHz	H 3, V 35	7 150
Humminbird 360	Surface	No	Unknown	*	Unknown	Unknown	2 400

Commercially available logging solutions was found to be expensive without being able to provide the power savings strategies needed for long deployments. For this reason, the approach of a building a custom logger was deemed needed and the ease of integration of each candidate sonar was taken into consideration.

In addition to the Echologger RS900, the Humminbird360 recreational fishing sonar was also investigated because of its low cost. However, integration was deemed not feasible while frequency and beam angles were unknown. Other low-cost alternatives like the Blue Robotics Ping360 and Tritec Micron simply did not have the required resolution to observe smaller features and are better suited for obstacle avoidance.

### 2.3 Controller

A controller manages the data acquisition as well as the power management of the system. These two subsystems are each run by a commercially available and community-supported controller. The controller stack connects to the sonar, an on/off dongle and a magnetic switch, which is used to determine the mode of the sonar controller.

#### 2.3.1 Power controller

For the power controller (named “Barnacle” during development), the ATMeage328p was selected as it was widely available as the popular Arduino Pro Mini board (and the commercial copies of this board), which benefits from a lower clock speed (8 MHz) and the absence of a USB to serial chip to reduce overall power consumption. The primary method of reducing power consumption in the system was to power down all non-essential systems during sleep and to act as an intervalometer: a device that trigger on a set interval. This sleep state is configured via a micro SD card. It is worthwhile mentioning that between the development of this hardware and the writing of the paper, the commercialization of this specific controller board has been discontinued and no longer in production by the official manufacturer. Nevertheless, the microcontroller chip and “clone” boards remain available on the market.

#### 2.3.2 Sonar controller

The interaction with the sonar and the data acquisition is handled by a second controller (named “Baleen”) which holds a Raspberry Pi Zero W (selected for lower power consumption), an RS232 to UART converter chip to facilitate communications with the sensor, two power switches, and real-time clock, for accurate time keeping and data timestamping. The RS232 and power switches make up two separate interfaces which can be used for communication and powering on/off devices. One of these interfaces powers up when the system wakes, the second is connected to an IO pin of the Pi. In the current configuration, one interface is used to power the sensor before communication with the sonar is established on the second interface. As only one sensor is used this can be changed to accommodate a second sensor in the future.

The manufacturer was able to provide an integration manual which allowed for quick interfacing with the sensors.

**Fig. 3:**
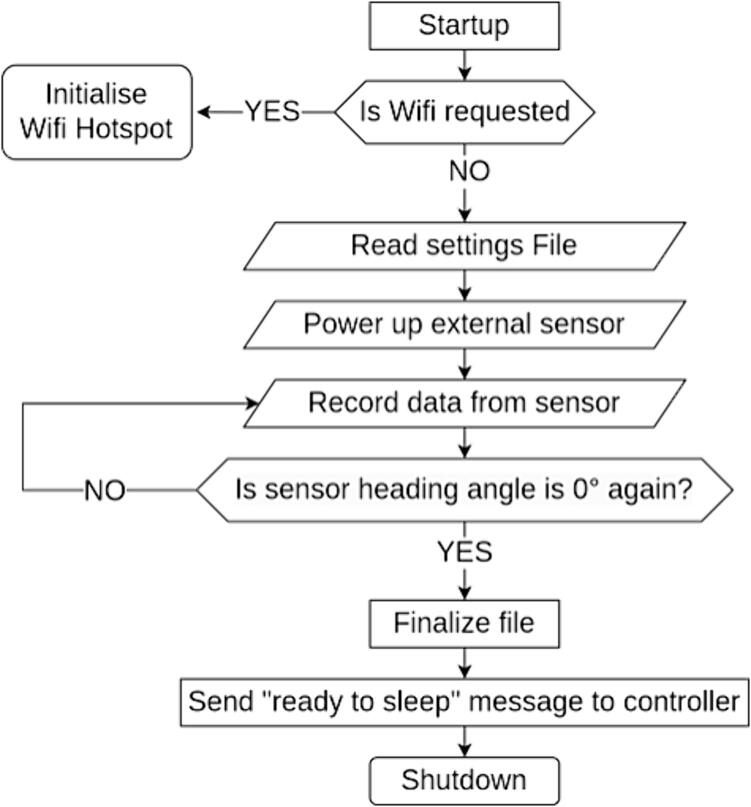
Raspberry Pi interfacing software logic flow.

The WI-FI capability of the Raspberry Pi Zero W supports the download of the recorded data subsequent to the deployment, as well as the application of changes to the recording settings file after the housing has been sealed. This WI-FI mode is off by default for power requirements, but can be activated with a magnetic switch, which generates an input signal that the Pi reads on start-up and starts the system as a wireless access point from where the data can be downloaded and new settings files can be uploaded. Fig. 3 summarises the Raspberry’s software logic flow.

### 2.4 Underwater housing

A custom underwater housing for a universal battery pack was designed and manufactured. This in-house approach was taken in attempt to compete with commercial products. The battery housing manufacturing cost, including material costs and labour, but excluding the underwater connectors and fasteners, came to 825 EUR. More than half of this cost can be attributed to labour. Please see Table 8 for a breakdown of the material costs. An off-the-shelf battery pack housing from Nortek costs around 1500 EUR with a single connector. Other options such as BlueRobotics housings provide further cost reductions with reduced inner diameters. The benefit of the custom housing is that the size and diameter were specifically designed to accommodate the controller stack and a custom battery pack with different existing battery cell technologies in an efficient manner with a viable minimum diameter. This diameter was determined based on an optimal D-Cell arrangement of up to 9 cells. Specifically, commercially available D-Cells was chosen as the primary cell to use in the battery pack because of their availability and the variety of battery chemistries that have adopted this form factor.

Furthermore, the housing was designed as a simple, rugged cylinder, made from POM (polyoxymethylene) with two endcaps containing double O-ring seals, for redundancy against water and sediment ingress. One of the endcaps was fitted with 2 commercial underwater connectors. The housing was designed to work at depths up to 500 m. Simulations have shown that the crush depth of the housing is around 885 m. Selecting a safety factor of 1.5, a safe working depth of 590 m can be assumed. This however needs to be validated through physical pressure testing. For the planned Sonarlogger deployments in depth no greater than 40 m, which equates to a safety factor of over 22, this was deemed adequate. The final housing dimensions are 480 mm (length), 160 mm (outer diameter), and 131 mm (inner diameter).

**Fig. 4:**
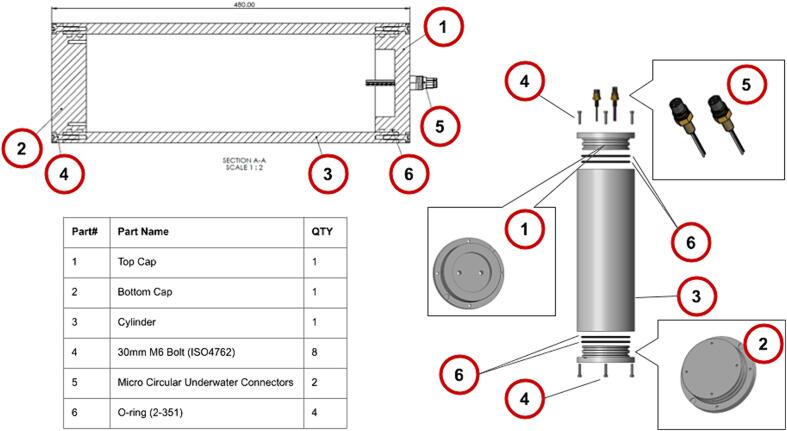
Underwater housing parts.

To connect the internal electronics of the underwater housing to the sonar and the on/off input, two commercial underwater connectors were used. For this, two holes were drilled and tapped for the connectors. In Fig. 4 an overview is given of the parts of the underwater housing assembly.

### 2.5 Battery pack

**Fig. 5:**
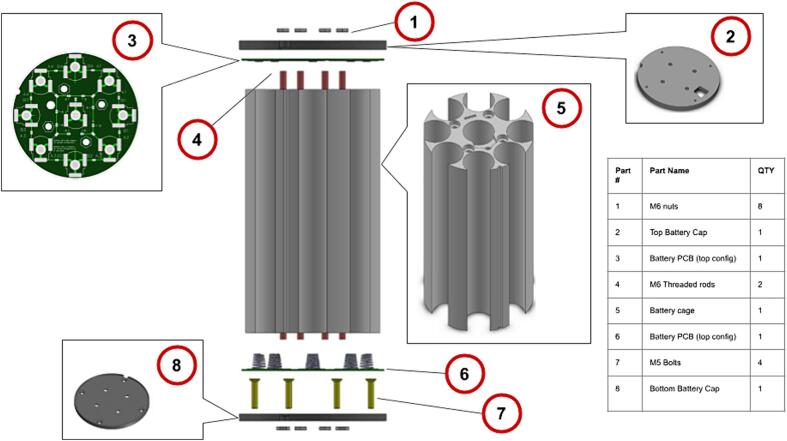
Parts in internal battery pack assembly.

The battery pack of the system was designed with versatility in mind. A D-Cell battery cage was designed to be 3D-printed to allow easy scaling depending on the battery requirements. This battery cage was printed from cost effective polylactic acid (PLA). To make the electrical connection between the cells a battery connector PCB was designed. This allows for customization of the battery cell configuration as this PCB can be set up with a series of solder jumpers, according to the desired cell configuration, for both series and parallel cell configurations. To mechanically hold the assembly in place under the tension from the spring terminals of the battery connector plates, a top and bottom battery cap was 3D printed. These two caps are held together by four threaded rods and captive low-profile nuts on the bottom battery cap. Fig. 5 shows the parts of the battery pack assembly.

D-Cells were selected as the most versatile battery format for this first build. Various battery chemistries are available in the D-Cell format and can be selected depending on the application; from rechargeable nickel-metal hydride (NiMH) batteries to lithium (where shipping restrictions allow) and readily available alkaline batteries.

The battery pack configuration used in this project is in an 8 series and 4 parallel (8S4P) arrangement of industrial alkaline D-Cell cells. This provides total pack voltage of 12 V as each cell provides a nominal 1.5 V. The total pack voltage is calculated as 8 cells in series x 1.5 V = 12 V. Nominal capacity for the specific cells (Proline D-Cell) is stated as 19 669 mAh, however, 15 000 mAh was assumed for the capacity estimation. Capacity of these cells can vary between brands from 12 000 mAh to 18 000 mAh. The total pack Ah is calculated as: 4 cells in parallel x 15 000 mAh = 60 000 mAh. The primary sensor, the sonar, operates on a voltage range of 12 V–72 V. This means that additional electronics is needed in the form of a step-up voltage converter, to prevent the sonar from attempting to power up when the pack voltages dips below 12 V during use. Alternatively, a 15 V pack configuration could be made in a 10S configuration, but this will reduce the capacity and will still need additional electronics to operate below 50 % of the back capacity. The step-up voltage converter selected for this was the Pololu U3V70F15, which can convert voltages to 15 V from well below the packs “depleted” state of about 7.2 V.

Section 6.1.1 further elaborates on the endurance estimation.

As the battery pack can be configured to take other battery technologies, the alkaline pack can be compared to a lithium primary configuration and a nickel metal hydride rechargeable option (compared in Table 4).

**Table 4:** Battery technology comparisons.

**Battery Parameters**	**12 V Alkaline Pack**	**14.4 V Lithium Pack**	**9.6 V NiMH Pack**
**Battery technology**	Alkaline D-Cells	Lithium D-Cells	NiMH D-Cells
**Cell voltage (V)**	1.5	3.6	1.2
**Cells in series**	8	4	8
**Cells stacks in parallel**	4	9	4
**Battery pack configuration**	8S4P	4S9P	8S4P
**Single cell capacity (mAh)**	15 000	17 000	8 500
**Total number of cells**	32	36	32
**Price per cell (Euro)**	3.0	26.0	10.0
**Price per pack (Euro)**	96.0	936.0	320.0
**Batt Pack Voltage (V)**	12	14.4	9.6
**Pack capacity (mAh)**	60 000	153 000	34 000

## 3 Design Files summary

All design files are available on the project OSF repository: https://doi.org/10.17605/OSF.IO/4GTZ8. (See Table 5, Table 6, Table 7)

**Table 5:** Mechanical Drawings design files as found in the OSF repository.

**Design file name**	**File type**	**Open-source license**	**Location of the file**
**Housing**			
*BottomCap_CAD*	CAD STEP file	CC BY 4.0	https://osf.io/mvyne
*BottomCap*	PDF	CC BY 4.0	https://osf.io/4e7v3
*BottomCap_SLDWKS*	Solidworks part	CC BY 4.0	https://osf.io/hyd6s
*TopCap_CAD*	CAD STEP file	CC BY 4.0	https://osf.io/4n59d
*TopCap*	PDF	CC BY 4.0	https://osf.io/5fsxm
*TopCap_SLDWKS*	Solidworks part	CC BY 4.0	https://osf.io/b3wst
*Cylinder_CAD*	CAD STEP file	CC BY 4.0	https://osf.io/z9g7m
*Cylinder*	PDF	CC BY 4.0	https://osf.io/s6q5h
*Cylinder_SLDWKS*	Solidworks part	CC BY 4.0	https://osf.io/749tc

**Battery Pack**			
*BatteryCage*	CAD STL file	CC BY 4.0	https://osf.io/k4agn
*BatterCage_SLDWKS*	Solidworks part	CC BY 4.0	https://osf.io/53z4x
*BottomBatteryCap*	CAD STL file	CC BY 4.0	https://osf.io/tzdy6
*BottomBatteryCap_SLDWKS*	Solidworks part	CC BY 4.0	https://osf.io/3xk7q
*TopBatteryCap*	CAD STL file	CC BY 4.0	https://osf.io/3brzm
*TopBatteryCap_SLDWKS*	Solidworks part	CC BY 4.0	https://osf.io/7pm94
*MountingPlate*	DXF	CC BY 4.0	https://osf.io/6nz7s
*MagneticSwitch*	DXF	CC BY 4.0	https://osf.io/brf57
*MagneticSwitch_CAD*	CAD STEP file	CC BY 4.0	https://osf.io/j3fua

**Table 6:** Electronic schematics and PCB design files as found in the OSF repository.

**Design file name**	**File type**	**Open-source license**	**Location of the file**
SONARLOGGER_BOM	CSV	CC BY 4.0	https://osf.io/4m5gy

**Power Controller**			
*Barnacle_sch_JSON*	EasyEDA JSON	CC BY 4.0	https://osf.io/a2my5
*Barnacle_sch_PDF*	PDF	CC BY 4.0	https://osf.io/srb2n
*Barnacle_PCB*	Gerber	CC BY 4.0	https://osf.io/smwk3
*Barnacle_PCB_JSON*	EasyEDA JSON	CC BY 4.0	https://osf.io/9an6x
*Barnacle_PCB_PDF*	PDF	CC BY 4.0	https://osf.io/eykm2
*Barnacle_BOM*	CSV	CC BY 4.0	https://osf.io/syjkc

**Sonar Controller**			
*Baleen_sch_JSON*	EasyEDA JSON	CC BY 4.0	https://osf.io/gdqzs
*Baleen_sch_PDF*	PDF	CC BY 4.0	https://osf.io/4pgdw
*Baleen_PCB*	Gerber	CC BY 4.0	https://osf.io/6tnxy
*Baleen_PCB_JSON*	EasyEDA JSON	CC BY 4.0	https://osf.io/83rgb
*Baleen_PCB_PDF*	PDF	CC BY 4.0	https://osf.io/r8vy2
*Baleen_BOM*	CSV	CC BY 4.0	https://osf.io/75jn4

**Battery Connector**			
*BatteryConnector_sch_JSON*	EasyEDA JSON	CC BY 4.0	https://osf.io/8pme6
*BatteryConnector_sch_PDF*	PDF	CC BY 4.0	https://osf.io/yzvte
*BatteryConnector_PCB*	Gerber	CC BY 4.0	https://osf.io/uzfhd
*BatteryConnector_PCB_JSON*	EasyEDA JSON	CC BY 4.0	https://osf.io/5xbhc
*BatteryConnector_PCB_PDF*	PDF	CC BY 4.0	https://osf.io/ja5bp
*BatteryConnector_BOM*	CSV	CC BY 4.0	https://osf.io/gh7tm

**Table 7:** Software and Firmware design files as found in OSF repository.

**Design file name**	**File type**	**Open-source license**	**Location of the file**
**Controller Firmware**			
*main.cpp*	C++	CC BY 4.0	https://osf.io/6dg5n
*Sonarlogger-CMDFILE-Reference*	PDF	CC BY 4.0	https://osf.io/qjax3
*Cmdfile*	Text file	CC BY 4.0	https://osf.io/6jga9

**Logger software**			
*main.py*	Python script	CC BY 4.0	https://osf.io/ra9jg
*settings.ini*	Text file	CC BY 4.0	https://osf.io/gjakr
*sonarlogger.service*	Text file	CC BY 4.0	https://osf.io/ayjpw
*SonarLogger.img*	Disk Image	CC BY 4.0	https://osf.io/buxz7

**Processing**			
*sonarplotter.py*	Python script	CC BY 4.0	https://osf.io/g378h
*makeVideo.sh*	Shell script	CC BY 4.0	https://osf.io/zxuhj
*bin2XFT.py*	Python script	CC BY 4.0	https://osf.io/hnjdy

## 4 Bill of materials

The bill of materials of each subsection of this build is provided with the design files of each component on the OSF repository of this project. This can be reached on: https://doi.org/10.17605/OSF.IO/4GTZ8. In Table 8 an overview bill of materials (BOM) is given. Note that each controller has an additional BOM, referenced in Table 8. Please notes that unit prices are calculated from minimum order quantities and prices exclude labour and shipping costs.

**Table 8:** Overview bill of materials.

**#**	**Designator**	**Component**	**QTY**	**Cost per Unit**	**Total cost**	**Source of materials**	**Material type**
**Underwater housing:****https://osf.io/8udnc**							
1	Underwater housing	Cylinder	1	€214.00	€214.00	Manufactured	POM
2	Underwater housing	Top Cap	1	€63.50	€63.50	Manufactured	POM
3	Underwater housing	Bottom cap	1	€63.50	€63.50	Manufactured	POM
4	Underwater housing	Underwater connector (MCBH2F)	1	€150.00	€150.00	https://www.dwtekmarine.com/products/p/connector_microcircular_bulkhead	
5	Underwater housing	Underwater connector (MCBH6F)	1	€150.00	€150.00	https://www.dwtekmarine.com/products/p/connector_microcircular_bulkhead	
6	Underwater housing	O-Rings (2–351)	4	€2.75	€11.00	https://shop.eriks.nl/en/seals-o-rings-and-accessories-o-rings/o-ring-nbr-70-36624-as568-bs1806-iso3601-351-120-02x5-33mm-10027512-en/	NBR
7	Underwater housing	M6 x 30 mm Hex socket head screw	8	€0.29	€2.32	https://shop.eriks.nl/en/fasteners-bolts-socket-head-cap-screws-fillister-head-screws/din912-hex-socket-head-cap-screw-stainless-steel-a4-80-m6x30mm-23268476/	A4 Stainless Steel

**Battery pack:****https://osf.io/t74nj**							
8	Battery pack	Battery Cage	1	€106.80	€106.80	https://jlc3dp.com/3d-printing-quote	PLA/SLA
9	Battery pack	Bottom Battery Cap	1	€7.69	€7.69	https://jlc3dp.com/3d-printing-quote	SLA
10	Battery pack	Top Battery Cap	1	€8.98	€8.98	https://jlc3dp.com/3d-printing-quote	SLA
11	Battery pack	Battery connector PCB	2	€1.85	€3.70	https://jlcpcb.com/	PCB
12	Battery pack	Mounting Plate	1	€6.05	€6.05	https://kunststofplatenshop.nl/product/budget-plexiglas-helder-3-mm/	PCB/Acrylic
13	Battery pack	M6 Low profile nuts	8	€0.02	€0.16	https://shop.eriks.nl/en/fasteners-nuts-hexagonal-full-nuts/hex-nut-low-din439-steel-4-m6-23690150/	Steel
14	Battery pack	M6 x 300 mm Threaded rod	4	€1.16	€4.64	https://shop.eriks.nl/en/fasteners-threaded-rods-and-ends-threaded-rods/din975-8-8-m6-1-draadstang-metrisch-lengte-1m-23679433-en/	Steel
15	Battery pack	M5 x 25 mm bolts	4	€0.09	€0.36	https://shop.eriks.nl/en/fasteners-bolts-socket-head-cap-screws-countersunk-screw/din7991-iso10642-hex-socket-countersunk-screw-stainless-steel-a2-m5x25mm-23264002	Steel
16	Battery pack	Alkaline D-Cells	32	€3.36	€107.52	https://be.farnell.com/en-BE/procell/pc1300-con-b10/battery-alkaline-1-5v-10pk/dp/3927144	
17	Battery pack	D-Cell Terminals (Pos)	8	€0.68	€5.44	https://be.farnell.com/en-BE/keystone/5250/battery-contact-button-d-cell/dp/4049827	Steel
18	Battery pack	D-Cell Terminals (Neg)	8	€1.03	€8.24	https://be.farnell.com/en-BE/keystone/5251/battery-contact-spring-d-cell/dp/4049828	Steel
19	Battery pack	XT-60 connect Female	1	€0.45	€0.45	https://www.tinytronics.nl/en/cables-and-connectors/connectors/others/xt60u-connector-set	
20	Battery pack	XT-60 connect Male	1	€0.45	€0.45	https://www.tinytronics.nl/en/cables-and-connectors/connectors/others/xt60u-connector-set	
21	Battery pack	Reed switch	1	€0.40	€0.40	https://www.tinytronics.nl/en/switches/magnetic-switches/reed-relay-2*14mm	
22	Battery pack	Magnetic key	1	€6.05	€6.05	https://kunststofplatenshop.nl/product/budget-plexiglas-helder-3-mm/	Acrylic
23	Battery pack	10 mm magnet	1	€0.60	€0.60	https://www.tinytronics.nl/en/mechanics-and-actuators/parts/magnets/neodymium-magnet-2*10mm	Neodymium
**Controller****https://osf.io/pe7jz**							
24	Controller	Power Controller	1	€47.98	€47.98	Manufactured	PCB
25	Controller	Sonar Controller	1	€42.24	€42.42	Manufactured	PCB
26	Controller	15 V regulator (U3V70F15)	1	€20.69	€20.69	https://www.pololu.com/product/2896	
27	Controller	JST connector Male	2	€0.10	€0.20	https://www.tinytronics.nl/en/cables-and-connectors/connectors/jst-compatible/jst-ph-2p-compatible-pcb-connector-male	
28	Controller	XT-60 connect Male	1	€0.45	€0.45	https://www.tinytronics.nl/en/cables-and-connectors/connectors/others/xt60u-connector-set	
29	Controller	XT-60 connect Female	1	€0.45	€0.45	https://www.tinytronics.nl/en/cables-and-connectors/connectors/others/xt60u-connector-set	
30	Controller	M2.5 x 10 mm standoff (Spacer kit)	12	€8.00	€96.00	https://www.tinytronics.nl/index.php?route=product/search&search=M2.5	Brass
31	Controller	SD Cards (16 GB)	2	€7.75	€15.50	https://www.tinytronics.nl/en/sandisk-ultra-16gb-class-10-uhs-i-a1-microsd-card-with-sd-card-adapter	

**TOTAL COST**					€1,145.36		

## 5 Build instructions

### 5.1 Internal battery assembly

**Fig. 6:**
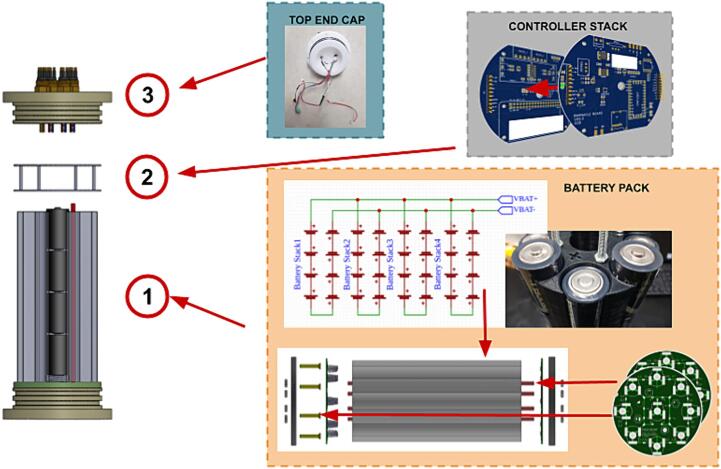
Parts in internal battery pack assembly.

The three primary mechanical parts of the internal assembly are the battery holding cage, and bottom and top plate that caps off the assembly, as seen in Fig. 6.

The internal battery pack is assembled as follows:

1. Assemble the two battery connector PCBs according to the desired configuration;
2. Make sure that the top battery connector PCB is soldered with two 10 mm wires going to a male XT-60 connector;
3. Lubricate the two O-rings and slide them onto the bottom end caps into their grooves. Care must be taken not to damage O-rings during this step;
4. Insert four low-profile M6 nuts in the recesses on the back of the bottom battery cap;
5. Screw the bottom battery cap to the bottom end cap of the underwater enclosure with the four M5 stainless steel screws;
6. Insert the bottom battery connector PCB with the nine spring contacts soldered to it;
7. Screw the four threaded rods in the captive nuts and add four low profile M6 nuts to tighten the PCB against the bottom plate;
8. Slide the 3D-printed cage over the four threaded rods until the cage contacts the bottom PCB;
9. Insert the 32 D-cells into the battery cage (see Fig. 6), alternating with four cells in positive alignment and four in negative alignment to give the 8S4P configuration;
10. Fit the top PCB (top configuration) in the top cap and put it on top of the battery pack closing the assembly. Make sure to align the slots on the three main parts;
11. Compress the battery spring contacts by adding four low profile M6 nuts pressing the top plate against the cage.

### 5.2 Controller stack and electronics assembly

**Fig. 7:**
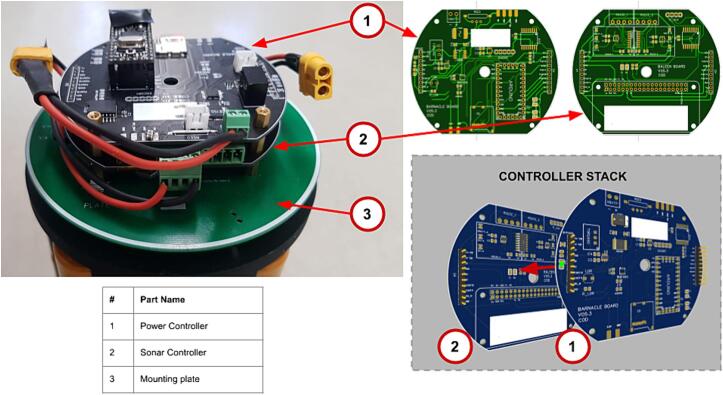
Parts in internal battery pack assembly.

Once the internal assembly has been completed, with the batteries installed, the controller stack can be installed. The controller stack consists of the Power Controller installed on top of the Sonar Controller as shown in Fig. 7.

This section will not explain how printed circuit boards are populated and will assume that the user can follow the schematic and PCB files provided in the repository, or, alternatively, has ordered the boards already populated.

In the design files, a drawing is given for a mounting plate, this facilitates the installation of the stack onto the battery pack.

The power controller board and sonar controller board stack on two 10 pin headers and are secured on four M3x10 mm standoffs on the electronics mounting plate.

**Fig. 8:**
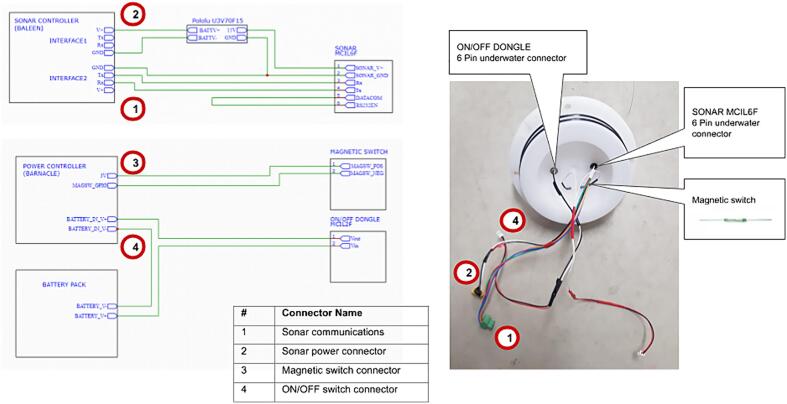
Connector identification and wiring.

In addition to this stack, the 15 V step-up voltage regulator (Pololu U3V70F15) is wired into the system (see Fig. 8) to ensure that the voltage supplied to the external sensor does not dip below 12 V as the alkaline pack depletes over the deployment. See section 2.5 for more information about the step-up voltage regulator.

The stack can be assembled as follows:

1. Install four M3x 10 mm standoffs on the mounting plate, with nuts on the bottom of the plate;
2. Install the mounting plate on top of the battery pack assembly, on the protruding threaded rods, fasten in place with low profile M6 nuts;
3. Install the sonar controller on top of the mounting plate, fastening it with four M3x 10 mm standoffs;
4. Stack power controller on top of the sonar controller via the 10 pin header pins. Make sure that the text is orientated the same on both boards;
5. Before proceeding, check that the none of the bottom board components are in contact with the top board. If this is found, consider covering the component with isolation or Kapton tape;
6. Fasten with four M3x 10 mm standoffs;
7. Install the 15 V step up regulator on the mounting plate;
8. Wire the input of the 15 V step up regulator to a 4way male WURTH 3.5 mm connector
9. Wire the output of the 15 V step up regulator to an XT-60 female connector.
10. Connect the WURTH male to the female connector on the sonar controller PCB
11. Connect the XT-60 male connector from the battery assembly to the XT-60 female connector

### 5.3 Wiring and connecting to the top end cap

The top end cap is the main interface for all external inputs and outputs to the logging device. This includes the sonar, the on/off dongle and the magnetic switch.

The top end cap has three wire assemblies:

1. The primary sensor cable enters via a 6-pin underwater connector (MCBH2F Impulse connector), which is then split into two connectors: Sonar Power (XT-60 connector) and Sonar Communications (Wurth 4 position 3.5 mm connector);
2. The ON/OFF switch from the 2-pin underwater connector (MCBH2F Impulse connector). These pins are shorted externally to power ON the device and start recording. This wiring internally goes to a JST connector;
3. The magnetic reed switch makes it possible to select the Wi-Fi mode for data recovery. This wiring internally goes to a JST connector.

The PCB stack is mounted on the electronics mounting plate and the cables can be connected to that end of the cap (cables and connections shown in Fig. 8).

Instructions for assembling the top end cap:

1. Screw the two underwater connectors into the top end cap. Be sure to take care that the O-rings of the connectors are seated correctly;
2. Solder two wires of 35 mm to the magnetic reed switch;
3. Hot glue the magnetic reed switch in-between the two connectors on the bottom side;
4. Solder wires of 30 mm to the two connectors wires
5. Solder these wires to their connectors as show in Fig. 8
  1. 6pin to four-way Wurth 3.5 mm and XT-60 connectors
  2. Magnetic switch to JST connector
  3. ON/OFF switch to JST connector

The ON/OFF dongle is made by simply shorting a 2-pin male underwater connector.

### 5.4 Housing final assembly

**Fig. 9:**
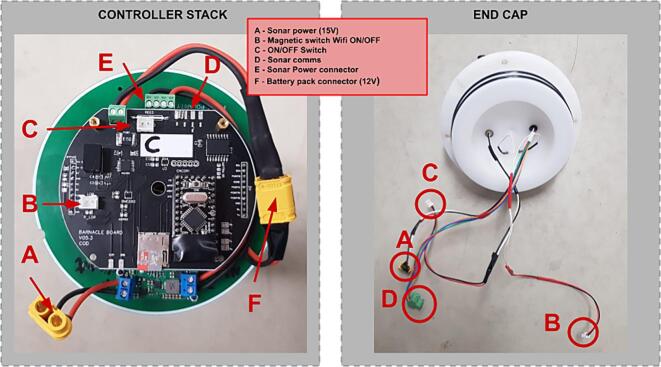
Connector identification.

The final assembly of the electronics into the underwater enclosure and the final sealing of the housing is described by the following steps (See Fig. 10 of the parts relevant to this section):

1. Lubricate the two O-rings and slide them onto the top end caps into their grooves. Care must be taken not to damage O-rings during this step;
2. Slide the tube over the battery assembly and firmly press the tube on the bottom end cap; Be sure to align the tube screw holes
3. From the top end cap, connect the wires from the battery/electronics with the wires from the connectors of the end cap, as assembled in Section 5.3. Connect as seen in Fig. 9;

**Fig. 10:**
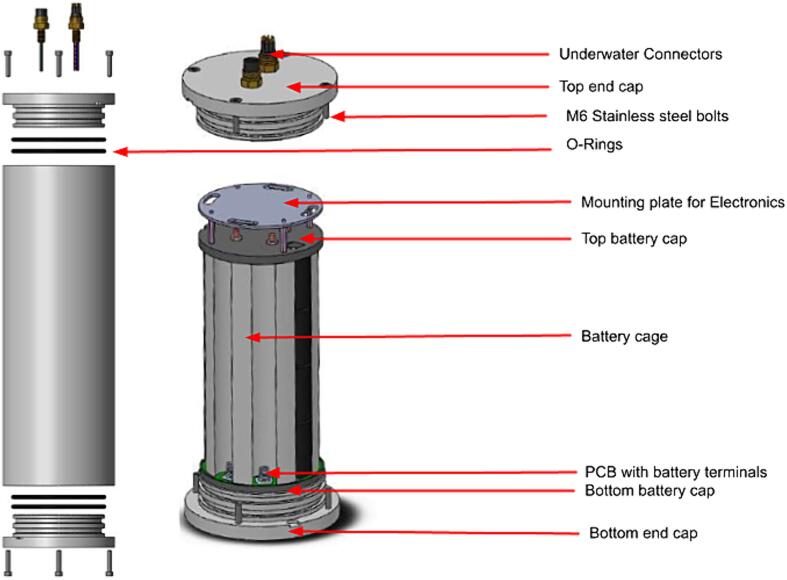
Final Assembly.
4. If possible, fit a desiccant bag into the remaining space. These are small bags filled with hygroscopic material that absorbs water and humidity to sustain a dry environment, protecting the battery or any other electronics from corrosion due to humidity in the air inside the enclosure.
5. Position the top end cap onto the tube, align the screw holes and firmly press down until the top end cap sits flush against the tube;
6. Finish by securing the endcaps to the tubes by screwing in the eight M6 screws.

## 6 Operation instructions

### 6.1 Pre-Deployment

#### 6.1.1 Endurance calculations

To calculate the estimated endurance of the system, some basic calculations and assumptions are made. The battery pack used in testing is in an eight series and four parallel (8S4P) configuration of industrial alkaline D-Cell cells. As stated in Section 2.5, the nominal pack capacity is around 60 000 mAh, 12 Volts.

The 360-degree sweep time of the sonar at the tested settings is about 10 min 30sec (limited by the maximum baud rate of the Raspberry Pi). Boot time and other processes add about another 5 min to the uptime at worst. This equates to maximum uptime of around 15 min. The Echologger datasheet from the manufacturer states the power draw as 6 W. As we are supplying the device with 15 V, we assume the current draw to be as 6 W / 15 V = 0.4 A. From bench measurements, however the practical average current draw as 300 mA and the sleep power consumption of the system is measured as ∼ 1.1 mA.

To calculate the total expected battery endurance, we can use Eq. (2).

(2) $$t_{\mathrm{en}}\;=\;\frac{B_{\mathrm{cap}}}{\left(\left(\frac{I_{\mathrm{active}}\times t_{\mathrm{active}}}{t_{\mathrm{active}}+t_{\mathrm{sleep}}}\right)+\left(\frac{I_{\mathrm{sleep}}\times t_{\mathrm{sleep}}}{t_{\mathrm{active}}+t_{\mathrm{sleep}}}\right)\right)\times \left(\frac{1-M_{\mathrm{safe}}}{100}\right)}$$

Where:

• t_en_ = Endurance in hours
• B_cap_ = Total battery pack endurance in Ah
• I_en_ = Current draw when active
• I_active_ = Current draw when active
• t_active_ = Time active
• I_sleep_ = Current draw when asleep
• t_sleep_ = Sleep time
• M_safe_ = Safety margin percentage

Table 9 has a comparison of a few different endurance possibilities when using different battery pack configurations.

**Table 9:** Battery pack endurance comparisons.

	**Actual**					
**Interval (hours)**	4	1	12	1	12	12
**Batt Pack Type**	Alkaline	Alkaline	Alkaline	Lithium	Lithium	NiMH
**Batt Pack (mAh)**	60 000	60 000	60 000	153 000	153 000	34 000
**Safety Margin (%)**	5	5	5	5	5	5
**Interval (min)**	240	60	720	60	720	720
**Awake Current Draw (mA)**	300	300	300	300	300	300
**Awake time (min)**	15.0	15.0	15.0	15.0	15.0	15.0
**Sleep Current Draw (mA)**	1.1	1.1	1.1	1.1	1.1	1.1
**Duty Cycle (%)**	5.882	20.000	2.041	20.000	2.041	2.041
**Battery life (hours)**	3230.984	950.836	9311.024	2423.336	23741.52	5276.690

**Battery life (days)**	134.624	39.618	387.959	100.972	989.230	219.862

It should be noted that there are many factors that can affect the operational endurance, from the brand of D-Cell, SD card used to the water temperature. The calculations above are meant as a best estimate for planning a deployment.

#### 6.1.2 Select sonar settings

To simplify changing common sonar configurations, the main python script references a settings file (settings.ini) where these parameters can be selected from a predetermined list. The settings file can be accessed on device with the same method described in Section 6.3. The settings file can be uploaded in the same fashion as the described process.

In the settings.ini file the user has access to the following parameters:

• Sweeps – Number of sweeps that the sonar will complete before going to sleep;
• Range – Radius of the sweep. Accepts the following ranges: 15, 20, 30, 45 m;
• Speed – The rotational speed of the sonar, slower gives better resolution. Accepts the following: slowest, slow, normal, fast, and fastest;
• Head – Set if the head is mounted upright or upside down;
• Rotation – Perform the sweep clockwise (cw) or counterclockwise (ccw).

Settings used in the tests of this project was as follows:

• Sweeps = 1
• Range = 30
• Speed= slow
• Head = up
• Rotation =cw

#### 6.1.3 Configuring the power management board

Setting the sleep interval is specified via a small text file (named CMDFILE) on the SD Card of the power management board. A description of the command file variables and how they are used are presented below in Table 10. The CMDFILE is a small text file that sets up the recording parameters. Note that when the SD Card or CMDFILE is not detected the device will revert to default values:

• interval = 240
• dusk_hour = 0
• dawn_hour = 0
• low_voltage = 0
• timeout = 180

**Table 10:** Description of the CMDFILE parameters.

**Variable**	**Description**
Interval	Interval is the time, in minutes, that the device will sleep for. It accepts only round integers. Set this value to 0 (zero) to keep the unit powered indefinitely. Example: interval = 2.

dusk_hour	The hour from which the controller should no longer wake up. After this, the device will wait for the “dawn_hour” to start its cycle again. This is typically used for defining “nighttime”. Set this value the same as the dawn_hour to ignore “nighttime” and keep recording around the clock. Example: dusk_hour = 20 (the device will not power up after 20:00, but wait for dawn_hour).

dawn_hour	The hour from which the controller can wake up. This is typically used for defining “nighttime”. Set this value the same as the dusk_hour to ignore “nighttime”. Example: dawn_hour = 5 (the device will power up after 05:00, until dusk_hour).

Low_voltage	This is the voltage at which the device will no longer power up to protect the battery pack from excessive discharge. For the 12 V alkaline battery pack, this is set as 9. Only accepts round numbers. Example: low_voltage = 9.

Timeout	The time in seconds that the controller will wait for a response from the data management board. Once this time is exceeded, the system will go back to sleep. Example: timeout = 180.

#### 6.1.4 Testing

Once configured and assembled, the system should be tested to be sure that all connectors are in place and all settings are accepted. Here follows a simple test procedure:

1. Connect the Echologger data cable and the ON/OFF dongle;
2. Leave this for 5–10 min;
3. Disconnect the ON/OFF dongle to power off the unit;
4. Follow the Downloading data instructions (Section 6.3.1) to check that the unit has generated new files: one “.bin” file and one “.log” file in the following formats:
  ○ auto_YYYYMMDD_HHMMSS.bin
  ○ auto_YYYYMMDD_HHMMSS.log

#### 6.1.5 Mounting considerations

The Echologger is provided with a mounting bar which allows the sonar to be clamped in a desired place with stainless steel hose clamps or tube clamps. Both options are readily available for many suppliers. For the test deployment, 30 mm tube clamps were selected.

**Fig. 11:**
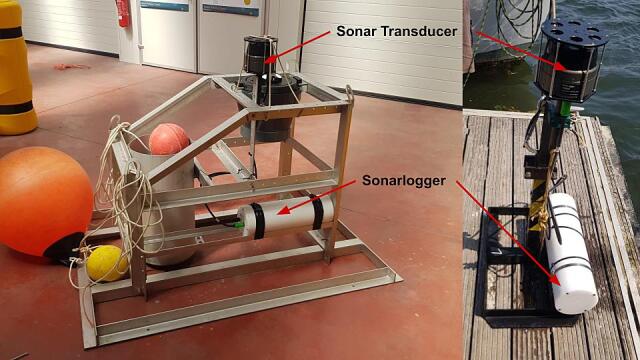
Mounting configurations during the testing phases.

**Fig. 12:**
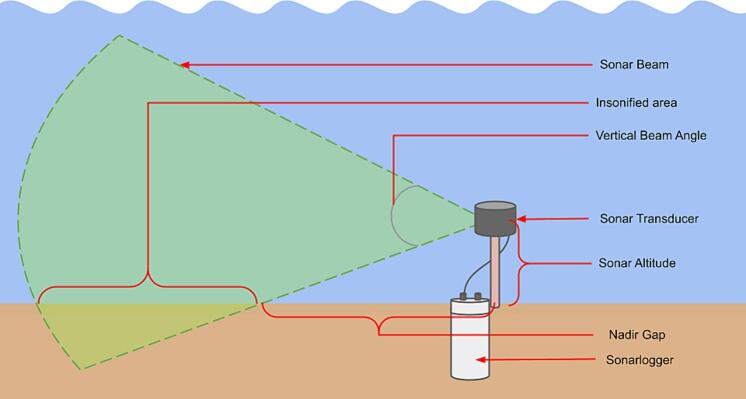
Diagram showing the measurement terminology during deployment.

It is important that the sonar transducer be mounted at a suitable height above the seafloor without any obstacles in view of the transducer beam (see Fig. 11). A height of about 1 to 1.5 m was found to provide good coverage of the target area based on testing done in the harbour and on the lander (See Fig. 11). This ensured that the structure, on which the sonar is mounted on, was in the Nadir gap (see section 6.2.1) and not obstructing the sonar beam. If the sonar head is too high off the seafloor, the ensonified area (see Fig. 12) will not be on the seafloor. If the sonar head is too low, the range can be reduced if there are sloping features.

Section 6.2.1 further elaborates on the considerations with regards to the sonar beam. Neither the sonar head nor the controller is able to provide orientation and heading information, so careful note should be taken of the deployment orientation.

### 6.2 Deployment

#### 6.2.1 Deployment considerations with regards to sonar characteristics

When planning the deployment, the sonar beam’s behaviour should be considered. Fig. 12 points out the terminology used in these considerations. To estimate the scan characteristics, we can use the following calculations. To determine the minimum range or Nadir as in Eq. (3):

(3) $$L_{\mathrm{nadir}}=\tan\left(90-\left(\frac{\emptyset_{v}}{2}\right)\right)\times h$$

Secondly, in Eq. (4), to determine the minimum depth needed to avoid surface reflections:

(4) $$d_{\mathrm{clear}}=\left(r\times sin\left(\frac{\emptyset_{v}}{2}\right)\right)+h$$

Thirdly, to determine the spatial resolution or physical space represented by a single sonar return, at a certain distance from the sonar head, as shown here in Eq. (5).

(5) $$W_{\mathrm{rtn}}=2\left(L\times sin\left(\frac{\emptyset_{h}}{2}\right)\right)$$

Where:

• $L_{\mathrm{nadir}}$ = Minimum distance of the sonar scan
• h = Height of the sonar transducer off the seafloor (altitude)
• r = Range setting of the sonar
• $d_{\mathrm{clear}}$ = Minimum depth needed to avoid surface reflections
• $W_{\mathrm{rtn}}$ = Width of a sonar return a target distance
• L = Distance from the sonar head to a target
• $\emptyset_{v}$ = Vertical beam angle of the sonar
• $\emptyset_{h}$ = Horizontal beam angle of the sonar

#### 6.2.2 Switching ON

Starting up the device requires that the two pins of the ON/OFF connector be connected to each other. This is done by a short ON/OFF “dongle” cable that simply shorts these pins.

The procedure to power up the device is as follows:

1. Be sure to remove the WI-FI-key;
2. Ensure that the sensor is connected;
3. Remove the dummy plug and store safely;
4. Insert the ON dongle, as shown in Fig. 13;

**Fig. 13:**
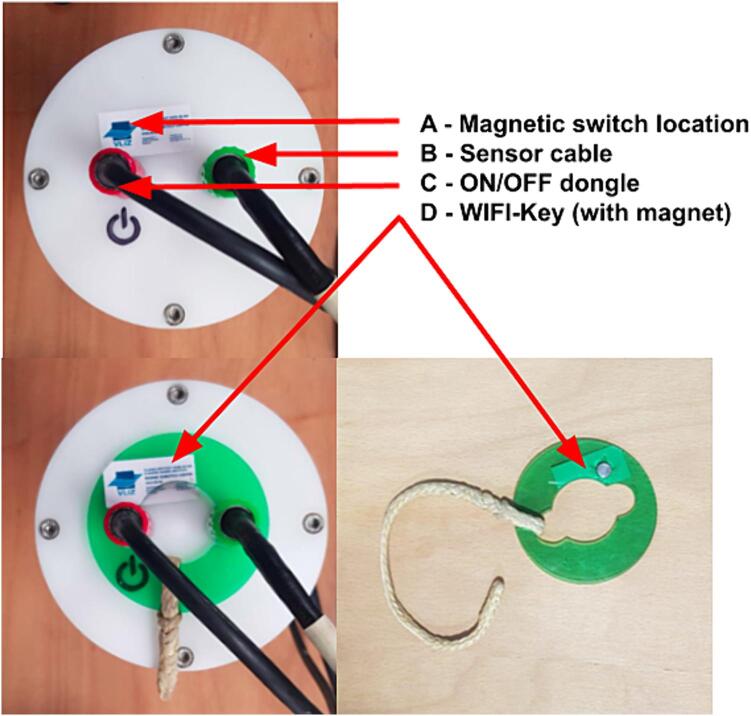
Top end cap with the green WI-FI key, on/off dongle and sensor connected. (For interpretation of the references to colour in this figure legend, the reader is referred to the web version of this article.)
5. Start-up can take 3–5 min.

The logger will immediately power-up and initialise the sensor to start recording. Expect this first data file to be recorded in air.

### 6.3 Recovery

#### 6.3.1 Data retrieval

As indicated in Fig. 13, the following steps for downloading data from the device over WI-FI should be adhered to:

1. Disconnect both connectors from the top end cap;
2. Place the WI-FI key as below. Note that the location of the magnetic switch is under the VLIZ sticker, so be sure to orientate the WI-FI key correctly;
3. Connect the cables;
4. Allow for ∼3–5 min for the WI-FI access point to start-up;
5. Check WI-FI for a new WI-FI connection named SONARLOGGER, connect with password Sonarlogger;
6. Open FileZilla and setup a connection as follows:
  a. IP: 192.168.1.10
  b. User: pi
  c. Pass: raspberry
7. Log in with the provided credentials;
8. Download all the.bin files. These are the sonar sweep data files;
9. Download all the.log files. These are the sweep logs;
10. Power down the system when done by removing the ON/OFF dongle;
11. Be sure to remove the WI-FI-key.

#### 6.3.2 Binary data conversion

A script is provided to convert the binary file into a XDF file, which enables the files to be processed in commercial sonar processing software such as SonarWiz.

#### 6.3.3 Data plotting

**Fig. 14:**
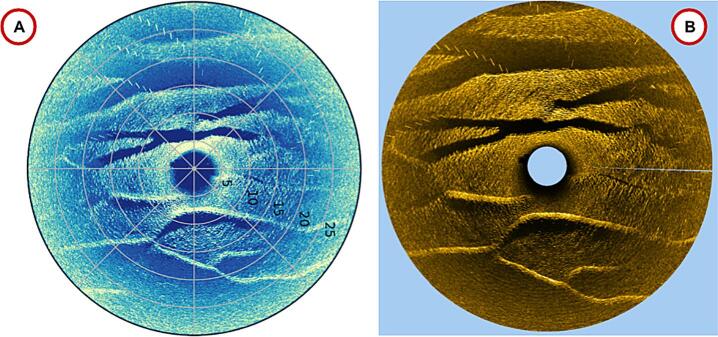
Output of the plotting script (A) and XDF plotted in SonarWiz (B).

Plotting of the data is done via a python script. The script accepts 3 arguments: the data file name, the rotation of the scan and the chosen colour map as seen in Fig. 14A.

Note that there is currently no compass heading recorded by the device, so the degrees are in reference to the “zero” location of the scanning sonar head.

## 7 Validation and characterization

### 7.1 Field tests

Five field tests were conducted with the system over the course of 2021 and 2022. For these deployments, the Sonarlogger was mounted on a multipurpose mooring. Total deployment time during these tests amounted to around 138 days, with the longest of these tests being 57 days. All tests were conducted in shallow waters of the Belgian Noth Sea, depths less than 30 m. The standard settings used during these tests was 4-hour intervals, 30 m sonar range at “slow” scanning speed. These settings were selected based on dock tests to find a balance between system uptime and data quality.

**Fig. 15:**
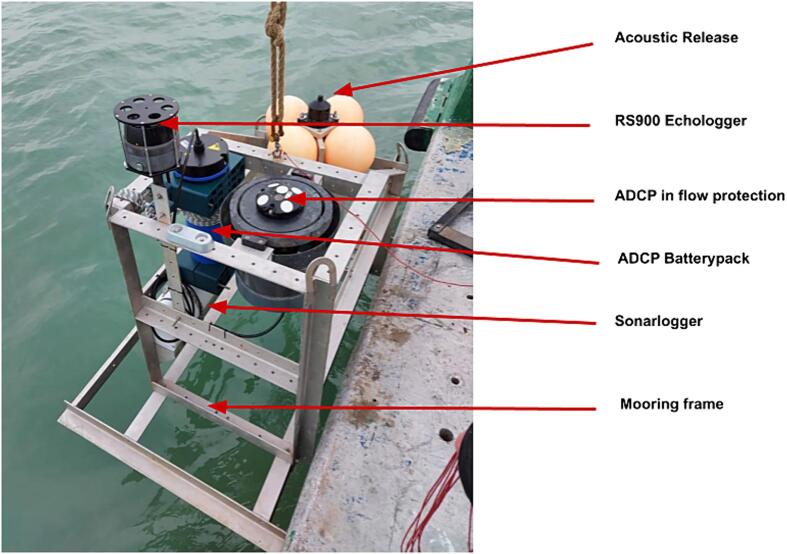
The Sonarlogger system mounted on a multipurpose mooring with an ADCP and acoustic release.

During these operations, the Sonarlogger was coupled with an ADCP, which was also mounted on the same mooring frame as shown in Fig. 15.

### 7.2 Results

The Sonarlogger was deployed from the 15th of February to the 12th of April 2023 at a depth of around 10 m in the Belgian North Sea. Over this time mussels from a decommissioned aquaculture installation were dumped into the target area of the sonar, as this was deemed the best possible analogue for a mussel reef as well as that it would have the same acoustic response off the shells.

**Fig. 16:**
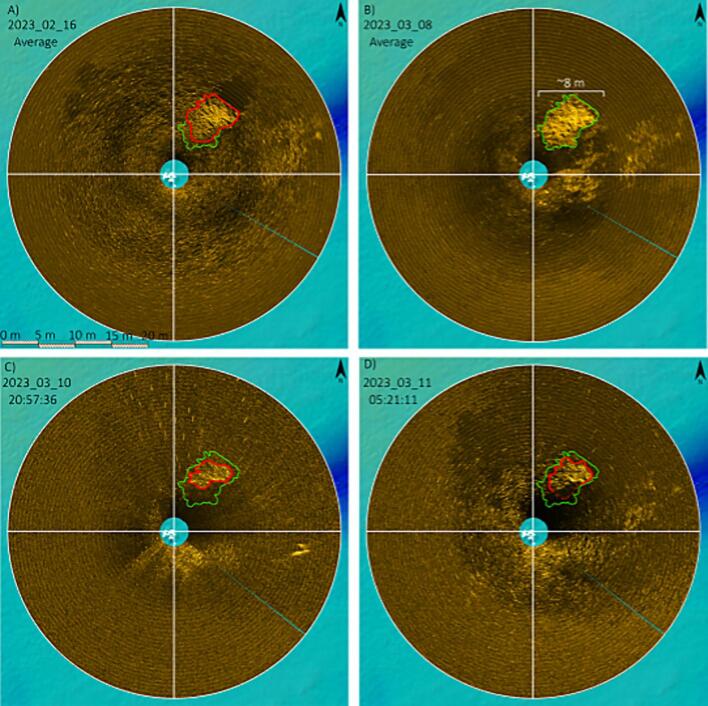
A series of scans of the seabed over the deployment period of 2023. Two mussel clumps are visible with the largest mussel clump highlighted on each image. The largest extent of the mussel clump is highlighted in green while the actual extent for each date is highlighted in red. (For interpretation of the references to colour in this figure legend, the reader is referred to the web version of this article.)

The main mussel dump (1800 kg) can clearly be seen in Fig. 16a, highlighted in red. After the dump the mussels appear to settle and spread across the sea floor, reaching a maximum size, highlighted in green (Fig. 16b). The second mussel clump can also be seen to the right of the main clump. The second mussel clump was located directly down current of the ebb stream. During the storm on the 10th of March 2023 the main mussel clump significantly reduced in size from the largest extent (Fig. 16c). Immediately after the storm mussel remained at the reduced size and the secondary mussel clump is also less visible. It should be noted that the mooring was shifted during the storm and was at an angle for the remainder of the deployment. This meant that part of the mussel clump was possibly no longer in the swath of the sonar. This shift was quantified by examining the data from the auxiliary sensor, which is this case was an ADCP with a compass.

The endurance of the system was validated by inspecting the voltage measured at the end of the deployment which was recorded as 10.2 V. Considering that the nominal “full” battery voltage is set to be 12 V (1.5 V × 8 cells in series) and the nominal “empty” battery voltage is 7.2 V (0.9 V × 8 cells in series), we can calculate the percentage of remaining capacity of the battery as follows: (10.2 V–7.2 V)/(12 V–7.2 V) × 100 = 62.5 %. This implies that only 37.5 % of the battery capacity was used. Considering that the total uptime for the system was 66 days we can deduce the projected endurance as: 66/(37.5/100) = 176 days. The discrepancy with the calculated estimated endurance of ∼134 days in Table 9 can be accounted for by the safety margins and pessimistic estimations of current draws.

On inspection of the system post deployment, no damages or leaks were seen. Inspection of the battery pack showed no mechanical damage internally. Data recording and timekeeping worked as expected and, although there was mild fouling on the transducer, the data quality was still adequate.

### 7.3 Potential improvements

Orientation sensors: Orientation measurements are not part of the current Sonarlogger package, but compass heading and orientation will aid in the processing of the data. Sensors like this will aid to the robustness of the data in the event of shift in positioning.

Further reductions in sleep power: Power hungry subsystems like SD Cards can be removed in future designs to reduce the overall sleep draw of the system, further increasing the endurance of the system.

Timing: The system currently sleeps on a set interval; it could be considered to allow the sweeps to be performed at specific times and tidal conditions.

Auxiliary sensors: Adding additional sensor measurements to the sonar sweeps can provide important context to the data that has been recorded. It would be possible to integrate an addition RS232 interface into the system for this, as the current controller design accommodates second sensor.

### 7.4 Conclusion

Stationary scanning sonar applications are not novel [2], [10], but their use has not been seen widely in long-term observations. The Sonarlogger system has been developed to address this gap. The Sonarlogger is a low-power logging system that integrates a stationary, high-resolution scanning sonar of the seabed. It can be deployed for extended periods of time, up to one year on a single battery pack, depending on the scan interval and settings. This long endurance capability reduces costs in comparison to boat-based sonar surveys over the same amount of time. The Sonarlogger is open-source, meaning the design files, instructions, and software are available to anyone who wants to build or modify the system or any of the subsystems.

Field tests have shown that the Sonarlogger can be successfully deployed in dynamic coastal environments. The system has been successfully used for over 130 days, over several deployments. The ability to operate independently of boats and divers, in any weather conditions, has the potential to provide a glimpse into the dynamics that affect biogenic reefs and seafloor features and processes.

To validate the sonars’ ability to observe a bivalve reef, mussels were collected from an aquaculture installation and dropped into the deployment site.

Preliminary results shown in Fig. 16 prove the applicability of the Sonarlogger system. Nevertheless, for a complete study of mussel reef dynamics (or other seabed feature), a further study based on longer deployments is required to determine the scales of changes that are observable, both in the spatial and temporal ranges.

Surveys at this time scale would not have been feasible in the form of vessel-based sonar surveys or diver surveys. Adverse weather would not have allowed a vessel to approach the site safely and the cost of the repeated surveys would have been expensive. In the context of the deployment discussed in the results, where the Sonarlogger provided 6 scans a day for 57 days, the cost for vessel charter at the nearest port was 2500–3300 euros (depending on the vessel) per day, excluding the cost of access to an appropriate sonar. However, the initial financial investment into any sonar technology remains expensive. In other deployment areas, where storms and visibility are less prohibitive, diver surveys may in fact be more cost effective.

The combination of the Sonarlogger with other environmental sensors can give a more detailed view into changes that affect the seafloor. Future work will focus on developing methods for using the Sonarlogger to collect data from a wider range of environmental conditions and on integrating additional sensors, such as ADCPs, CTDs, orientation sensors, and turbidity sensors.

## Ethics Statements

All contributors and participants are informed.

## CRediT authorship contribution statement

**Frederik-Willem Fourie:** Writing – review & editing, Writing – original draft, Visualization, Software, Resources, Methodology, Investigation, Conceptualization. **Kobus Langedock:** Visualization, Validation, Supervision, Project administration, Methodology, Investigation, Data curation, Conceptualization. **Roeland Develter:** Writing – review & editing, Visualization, Software, Resources. **Harold Loop:** Resources, Methodology, Investigation, Conceptualization. **Christopher J. Peck:** Writing – original draft, Visualization, Validation, Formal analysis. **Leandro Ponsoni:** Writing – review & editing, Writing – original draft, Validation, Supervision. **Hans Pirlet:** Writing – original draft, Supervision. **Wieter Boone:** Writing – original draft, Validation, Supervision, Project administration, Methodology, Investigation, Funding acquisition, Formal analysis, Data curation, Conceptualization.

## Declaration of competing interest

The authors declare that they have no known competing financial interests or personal relationships that could have appeared to influence the work reported in this paper.

## References

[1] Brown C.J. (2011). Benthic habitat mapping: A review of progress towards improved understanding of the spatial ecology of the seafloor using acoustic techniques. Estuar. Coast. Shelf Sci..

[2] Smith L. (2023). Relating side scan sonar backscatter data to geotechnical properties for the investigation of surficial seabed sediments. Geo-Mar. Lett..

[3] Purser A. (2022). A vast icefish breeding colony discovered in the Antarctic. Curr. Biol..

[4] Degraer S. (2008). Very-high resolution side-scan sonar mapping of biogenic reefs of the tube-worm Lanice conchilega. Remote Sens. Environ..

[5] Goedefroo N. (2022). Nature-based solutions in a sandy foreshore: A biological assessment of a longline mussel aquaculture technique to establish subtidal reefs. Ecol. Eng..

[6] Powers J. (2014). Evaluating the use of side-scan sonar for detecting freshwater mussel beds in turbid river environments. Hydrobiology.

[7] Ricklefs K. (2020). Occurrence, stability, and associated species of subtidal mussel beds in the North Frisian Wadden Sea (German North Sea Coast). Estuar. Coast. Shelf Sci..

[8] Greene A. (2018). Side scan sonar: A cost-efficient alternative method for measuring seagrass cover in shallow environments. Estuar. Coast. Shelf Sci..

[9] Montero-Hidalgo M. (2023). Mapping and assessing seagrass meadows changes and blue carbon under past, current, and future scenarios. Sci. Total Environ..

[10] Irish J.D. (1999). A self-contained sector-scanning sonar for bottom roughness observations as part of sediment transport studies. J. Atmos. Oceanic Technol..

